# Molecular identification of *Borrelia* spirochetes in questing *Ixodes ricinus* from northwestern Spain

**DOI:** 10.1186/s13071-017-2574-x

**Published:** 2017-12-20

**Authors:** Pablo Díaz, Jose Luis Arnal, Susana Remesar, Ana Pérez-Creo, José Manuel Venzal, María Esther Vázquez-López, Alberto Prieto, Gonzalo Fernández, Ceferino Manuel López, Rosario Panadero, Alfredo Benito, Pablo Díez-Baños, Patrocinio Morrondo

**Affiliations:** 1 Departamento de Patología Animal (Grupo INVESAGA), Facultad de Veterinaria, Universidade de Santiago de Compostela, Lugo, Spain; 2 Exopol, Zaragoza, Spain; 3 Laboratorio de Vectores y enfermedades transmitidas, Facultad de Veterinaria, CENUR Litoral Norte, Universidad de la República, Salto, Uruguay; 4 Servicio de Pediatría, Hospital Universitario Lucus Augusti, Lugo, Spain

**Keywords:** *Borrelia burgdorferi* (*s.l.*), *Borrelia miyamotoi*, *Ixodes ricinus*, NW Spain

## Abstract

**Background:**

*Ixodes ricinus*, the predominant tick species in Europe, can transmit the causative agents of important human diseases such as Lyme borreliosis (LB), caused by *Borrelia* spirochetes*.* In northern Spain, LB is considered endemic; recently, a significant increase of the annual incidence of LB was reported in the northwestern (NW) region.

**Methods:**

In order to provide information on the prevalence of *Borrelia* spp., pooled and individually free-living *I. ricinus* from NW Spain were molecularly analyzed. Positive samples were characterized at the *fla* and *Glpq* genes and the *rrfA-rrlB* intergenic spacer region to identify *Borrelia* species/genospecies.

**Results:**

*Borrelia burgdorferi* (*sensu lato*) (*s.l.*) individual prevalence and MIR were significantly higher in adult females (32.3 and 16%) than in nymphs (18.8 and 6.2%) and adult males (15.6 and 8.4%). Five *Borrelia* genospecies belonging to the *B. burgdorferi* (*s.l.*) group were identified: *B. garinii* was predominant, followed by *B. valaisiana*, *B. lusitaniae*, *B. afzelii* and *B. burgdorferi* (*sensu stricto*) (*s.s.*)*.* One species belonging to the tick-borne relapsing fever group (*B. miyamotoi*) was also found, showing low individual prevalence (1%), positive pool (0.7%) and MIR (0.1%) values. To our knowledge, this is the first citation of *B. miyamotoi* in free-living ticks from Spain.

**Conclusions:**

The significant prevalences of *B. burgdorferi* (*s.l.*) genospecies detected in questing ticks from NW Spain are similar to those detected in northern and central European countries and higher to those previously found in Spain. These results together with the high incidence of LB in humans and the high seroprevalence of *B. burgdorferi* (*s.l.*) in roe deer shown in other studies reveal that the northwest area is one of the most risky regions for acquiring LB in Spain.

**Electronic supplementary material:**

The online version of this article (10.1186/s13071-017-2574-x) contains supplementary material, which is available to authorized users.

## Background

*Ixodes ricinus* is considered the tick species with the widest distribution in Europe, transmitting the causative agents of important human and animal diseases; *Borrelia* spp. are amongst the most commonly identified pathogens in *I. ricinus* [1]. These spirochetes are clustered into three major phylogenetic groups, namely the Lyme borreliosis (LB) group, the relapsing fever (TBRF) group and the reptile-associated borreliae [2].

LB is caused by *Borrelia burgdorferi* (*sensu lato*) (*s.l.*); more than 20 *Borrelia* genospecies have been currently identified within the *B. burgdorferi* (*s.l.*) species complex. In Europe, LB is mainly caused by three genospecies: *Borrelia garinii*, associated with neuroborreliosis; *Borrelia afzelii* related to atrophic chronic acrodermatitis; and *Borrelia burgdorferi* (*sensu stricto*) (*s.s.*) related to arthritis [3]. *Borrelia bavariensis*, *Borrelia bissettii*, *Borrelia lusitaniae* and *Borrelia spielmanii* have been also identified as causal agents of LB in this continent [4]. Phylogenetically related to relapsing fever spirochetes, *Borrelia miyamotoi* has been generally identified worldwide in the same *Ixodes* species as *B. burgdorferi* (*s.l.*) [5, 6]. Although it was initially considered as non-pathogenic for people, human cases of *B. miyamotoi* disease (BMD), very different to relapsing fever cases, have recently been reported in the USA, the Netherlands, Russia and Japan [5, 7].

Twenty-four years ago, the overall annual incidence of LB in Spain was estimated to be 0.25 cases per 100,000 inhabitants although the disease is especially common in the northern regions where it is considered endemic [8]. In contrast, the endemic form of the TBRF has been diagnosed in central and southern regions, presenting a lower annual incidence (0.2/100,000 inhabitants) than LB [9, 10]. In Galicia (NW Spain), an increase of the annual incidence of LB was reported in recent years [11]. In addition, it was determined that roe deer from this region were highly exposed to *B. burgdorferi* (*s.l.*) [12]. However, currently no studies have been performed on the percentage of infection and molecular identification of *Borrelia* spp. isolated from ticks in NW Spain. The main aim of the present study was to identify the species/genospecies of LB and TBRF *Borrelia* in free-living *I. ricinus* from Galicia (NW Spain) and to provide information on their prevalence.

## Methods

### Study area

The study was performed in Galicia, a 29,574 km^2^ region located in the northwest of Spain (43°47′–41°49’N, 6°42′–9°18’W) with an oceanic climate, characterized by mild temperatures and high precipitation. Galicia is a major livestock breeding area, where animals are mainly reared in a semi-extensive management system; the landscape is composed of meadows (*Poa pratensis*, *Lolium perenne* and *Festuca pratensis*) and large autochthonous forest areas, the more common tree species being *Quercus robur* and *Castanea sativa* [13].

### Collection of ticks

In 2015, questing ticks were collected by flagging from vegetation in 17 different sampling locations. A 1 m^2^ piece of white cotton flannel was used and the ticks on the flag were counted and removed every 2 m. The flagging time (30 min of sampling) was similar in each site. Ticks were conserved in 90% ethanol until analyzed. In the laboratory, ticks were examined microscopically and identified to stage and species using different reference keys [14, 15].

Pools of ten whole *I. ricinus* nymphs or up to five adults from each sampling location were first analyzed in order to detect *Borrelia* spp. Data for the number of pools and ticks processed are summarized in Table 1. Initially, ticks were washed in sterile distilled water. Then, adult ticks were longitudinally cut with a sterile scalpel, and half only was used; the whole nymphs were processed. Finally DNA was extracted using a commercial kit (High Pure PCR Template Preparation Kit, Roche Diagnostics GmbH®, Mannheim, Germany) following the manufacturer’s instructions. In order to maximize DNA yields, ticks were first crushed with a micropestle in 200 μl of Tissue Lysis Buffer. All tick pools were subjected to a nested PCR targeting the *fla* gene, using previously described protocols [16, 17].

**Table 1:** Percentage of *Borrelia burgdorferi* (*s.l*.) and relapsing fever *Borrelia* positive individual ticks and pools, and minimum infection rate (MIR) in questing *I. ricinus* from NW Spain

		*Borrelia burgdorferi* (*s.l.*)	Relapsing fever *Borrelia*
Nymphs^a^	Positive ticks	26/192 (18.8%)	2/192 (1.0%)
	Positive pools	45/73 (61.6%)	0/73 (0)
	MIR	45/730 (6.2%)	0/730 (0)
Adult males^b^	Positive ticks	15/96 (15.6%)	1/96 (1.0%)
	Positive pools	12/32 (37.5%)	1/32 (3.1%)
	MIR	12/143 (8.4%)	1/143 (0.7%)
Adult females^b^	Positive ticks	31/96 (32.3%)	1/96 (1.0%)
	Positive pools	28/37 (75.7%)	0/37 (0)
	MIR	28/175 (16.0%)	0/175 (0)

^a^Pools of ten *I. ricinus*

^b^Pools up to five *I. ricinus*

In addition, the presence of *Borrelia* spp. DNA was determined from 384 (192 nymphs, 96 males and 96 females) individual *I. ricinus* samples by a real-time PCR assay. Adults and nymphs were processed and DNA extracted using the same protocol as for pool analysis. A qPCR targeting the flagellin gene (*fla*), especially developed for the present study, was designed manually based on multiple sequence alignment as follows: forward (5'-GCT CAA TAT AAC CAA ATG CAC ATG-3′), reverse (5′-AGA TTT GCA ACA TTA GCT GCA TAA A-3′) and probe (5′-/56-FAM/AAC AGC TGA AGA GCT TGG AAT GCA/3IABkFQ/-3′). The qPCR was performed using 5 μl of template into a 20 μl reaction final volume using GoTaq™ Master Mix (Promega, Madison, WI, USA) on a 7500 FAST cycler (Applied Biosystems, Foster City, CA, USA) under the following conditions: initial denaturation at 95 °C for 5 min, 40 cycles of denaturation at 95 °C for 15 s and annealing and extension at 60 °C for 1 min. To generate quantitative data, a synthetic oligonucleotide (Ultramer™, Integrated DNA Technologies Inc., Coralville, IO, USA) encompassing the *fla* region targeted by the primers was used as positive control. It was normalized at 5 × 10^5^ copies per reaction and 3 replicates from its serial 10-fold dilutions were tested to obtain efficiency, linearity and repeatability data of the assay. A cut-off value for positive samples was established at cycle quantification (Cq) values lower than 38. The inclusivity of this qPCR assay was tested analysing previously confirmed samples containing all *Borrelia burgdorferi* (*s.l.*) genospecies described in Spain [18–21]. All qPCR positive individual tick samples were also tested using the aforementioned *fla* nested-PCR. In order to identify *Borrelia* species/genospecies, PCR products were purified and subsequently sequenced; sequences were aligned and edited using ChromasPro (Technelysium, Brisbane, Australia), and consensus sequences were scanned against the GenBank database using the Basic Local Alignment Search Tool (BLAST; http://blast.ncbi.nlm.nih.gov/Blast.cgi). Finally, all individual and pooled samples identified as *B. burgdorferi* (*s.l.*) or *B. miyamotoi* were further characterized at the *rrfA-rrlB* intergenic spacer region (IGS) and the glycerophosphodiester phosphodiesterase (*GlpQ*) gene, respectively, using primers previously reported [22, 23]. Unique partial sequences identified in this study were deposited in the GenBank database under accession numbers MG245772–MG245790 and MG356949–MG356956 (Additional file 1: Table S1).

### Statistical analysis

The minimum infection rate (MIR) was used to estimate the prevalence of *Borrelia* in pooled ticks. This was calculated as the ratio of the number of positive pools to the total number of tested ticks, assuming that only one infected tick exists in a positive pool. Differences in both the individual prevalence and MIR between nymphs, adult males and adult females were analyzed by means of a Chi-square test. All statistical analyses were performed using IBM SPSS Statistics 20 (IBM Corporation, Armonk, New York, USA).

## Results

**Fig. 1:**
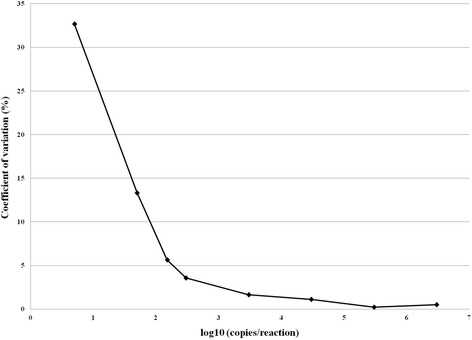
Intra-assay reproducibility of the qPCR targeting the flagellin gene (*fla* for the detection of *Borrelia* spp. DNA

The qPCR assay obtained 93.26% of efficiency and a linearity value of *R* ^2^ = 0.99. Moreover, the intra-assay reproducibility test resulted in a range of values from 0.5–32.7% (Fig. 1). The lower limit of detection was established as at least 5 copies per reaction and the lower limit of quantification was 50 copies per reaction. The inclusivity test resulted in positive detection of all genospecies mentioned before. Cq values ranged from 27.22 (5.14 × 10^3^ copies/reaction) to 37.87 (4.73 copies/reaction).

After *fla* and IGS PCRs, all positive samples were sequenced successfully, except for ten individual isolates which had underlying signals in the electropherogram that prevented the accurate readout of sequences. In both individual and pooled ticks, sequence analysis allowed the identification of four pathogenic *Borrelia* genospecies belonging to the *B. burgdorferi* (*s.l.*) group (*B. afzelii*, *B. garinii*, *B. lusitaniae* and *B. valaisiana*) and one belonging to the relapsing fever *Borrelia* group (*B. miyamotoi*); in addition, *B. burgdorferi* (*s.s.*) was only detected in pooled samples.

The prevalence of *B. burgdorferi* (*s.l.*) and relapsing fever *Borrelia* in both individual and pooled ticks as well as the minimum infection rate (MIR) are summarized in Table 1. The individual prevalence of *B. burgdorferi* (*s.l.*) was significantly higher in adult females than in nymphs (*χ* ^2^ = 14.174, *df* = 1, *P* < 0.001) and adult males (*χ* ^2^ = 7.319, *df* = 1, *P* = 0.006). In contrast, the mean number of *Borrelia* spirochetes per tick was higher in adult males (1.25e04; coefficient of variation (CV) = 2.19; range = 1.50e05–2.17e01) than in nymphs (4.73e03; CV = 2.67; 5.40e04–2.46e01) and adult females (2.83e03, CV = 2.55; 3.68e04–5.01e01). Considering the pooled samples, the percentage of positive pools was higher in both *I. ricinus* adult females and nymphs than in adult males (Table 1); furthermore, MIR was significantly higher in adult females than in nymphs (*χ* ^2^ = 18.635, *df* = 1, *P* < 0.001) and adult males (*χ* ^2^ = 4.143, *df* = 1, *P* = 0.042).

*B. miyamotoi* was only identified in four individual *I. ricinus* (two nymphs, one adult male and one adult female) and in one adult male pool, showing low individual and pooled prevalence and MIR values (Table 1).

In general, amongst all the six *Borrelia* species/genospecies detected, *B. garinii* was the predominant genospecies, followed by *B. valaisiana*, *B. lusitaniae* and *B. afzelii*. In contrast, *B. miyamotoi* and *B. burgdorferi* (*s.s.*) were only occasionally found (Table 2). Considering the different tick development stages, all five *Borrelia* species/genospecies detected in individual samples were identified in nymphs, males and females (Table 2). In contrast, five *Borrelia* species/genospecies were identified in nymph pools and four in adult male pools whilst only the three most frequent genospecies (*B. garinii*, *B. valaisiana* and *B. lusitaniae*) were detected in adult female pools (Table 2).

**Table 2:** Individual prevalence and minimum infection rate (MIR) of *Borrelia* genospecies of *Borrelia burgdorferi* (*s.l.*) and *Borrelia miyamotoi* considering the life stage of questing *I. ricinus* collected in NW Spain

	Nymphs		Adult males		Adult females	
	Individual	MIR	Individual	MIR	Individual	MIR
*Borrelia burgdorferi* (*s.l.*)						
*B. afzelii*	5/192 (2.6%)	2/730 (0.3%)	1/96 (1.0%)	1/143 (0.7%)	1/96 (1.0%)	0/175 (0)
*B. burgdorferi* (*s.s.*)	0/192 (0%)	1/730 (0.1%)	0/96 (0%)	0/143 (0)	0/96 (0%)	0/175 (0)
*B. garinii*	9/192 (4.7%)	25/730 (3.4%)	6/96 (6.3%)	8/143 (5.6%)	15/96 (15.6%)	13/175 (7.4%)
*B. lusitaniae*	1/192 (0.5%)	2/730 (0.3%)	3/96 (3.1%)	0/143 (0)	4/96 (4.2%)	5/175 (2.9%)
*B. valaisiana*	7/192 (3.6%)	15/730 (2.1%)	3/96 (3.1%)	3/143 (2.1%)	7/96 (7.3%)	10/175 (5.7%)
Relapsing fever *Borrelia*						
*B. miyamotoi*	2/192 (1.0%)	0/730 (0)	1/96 (1.0%)	1/143 (0.7%)	1/96 (1.0%)	0/175 (0)
Not identified *Borrelia*						
*Borrelia* spp.	4/192 (2.1%)	0/730 (0)	2/96 (2.1%)	0/143 (0)	4/96 (4.2%)	0/175 (0)

## Discussion

Our data revealed that *B. burgdorferi* (*s.l.*) is a very prevalent pathogen in questing *I. ricinus* from Galicia (NW Spain), with similar percentages to those recorded in some central and northern European countries [24–28]. The study area presents abundant *Borrelia*-reservoir and *I. ricinus*-maintenance host species (micromammals, birds, roe deer, wild boars, etc.), as well as proper habitat (deciduous forests) and climatic conditions (moderate precipitation and mild temperatures throughout the year) for questing ticks. In addition, our data showed values far higher than previous individual *B. burgdorferi* (*s.l.*) prevalences found in *I. ricinus* from other northern Spanish regions, where the percentage of infection did not exceed 9.3% [19, 21, 29]. This increment might be related to a recent increase in the prevalence of *B. burgdorferi* (*s.l.*) in free-living ticks, as recorded in some European countries in the last years [25, 30]. In fact, a significant increase of the annual incidence of human LB was observed in the study area, from 2.64 cases/100,000 inhabitants in 2007 to 11.61 in 2012 [11]. Changes in some biotic and abiotic factors might have favoured an increase in *I. ricinus* densities and consequently in *B. burgdorferi* (*s.l.*) prevalence; higher average temperatures recorded in the last years in NW Spain have led to milder winters that increased both the overwintering survival and the extension of the active period of free-living ticks and consequently extended the duration of the peak risk of exposure to tick-borne pathogens [31]. In addition, the progressive rural depopulation experienced by Galicia in recent decades has resulted in an increase of the extension of woodland areas, favouring the increase in range and abundance of *Borrelia*-reservoir hosts and maintenance hosts for *I. ricinus* [11].

*Borrelia burgdorferi* (*s.l.*) prevalence and MIR values were significantly highest in adult female ticks, which agrees with previous studies carried out in Europe [26, 32, 33]. *Ixodes ricinus* nymphs have a lower probability of being infected by *Borrelia* spp. than adults, which have ingested a supplementary potentially infected meal [34]. In addition, female nymphs consume more blood than male nymphs [35], which results in a higher probability to acquire bacteria.

Five *B. burgdorferi* (*s.l.*) genospecies were identified, confirming the wide *B. burgdorferi* (*s.l.*) genospecies diversity in ticks from Spain. *Borrelia garinii* and *B. valaisiana* were the predominant genospecies, which in general agrees with most studies carried out in Spain [18, 21]. Considering that *B. garinii* is mostly associated with neuroborreliosis, our results are in agreement with the high incidence of neurological symptoms observed in LB-infected patients from the same area of study [11, 36, 37]. *Borrelia garinii* was also identified in articular fluid from a patient with recurrent chronic arthritis on a knee [37]. An interesting finding was the occasional identification of *B. burgdorferi* (*s.s.*), even though it was reported as the predominant genospecies in some investigations performed in central and northern areas of Spain [20, 38]. Although *B. afzelii* was previously identified in a skin biopsy from a patient with chronic erythema migrans in northwestern Spain [36], it is worth noting that the detection of this *Borrelia* genospecies was not frequent in ticks of the study area. In addition, its significance in other Spanish regions was also limited [18, 20, 38]. Nevertheless, it was reported as one of the most common *Borrelia* genospecies in some central and northern European countries [39]. In Europe, the differences in geographical distribution of most *B. burgdorferi* (*s.l.*) genospecies were reported to be mainly related to an adaptation to infect particular groups of vertebrates, such as mammalian or avian hosts, but not to both, limiting transmission between different host species [39, 40]. In fact, *B. afzelii* has been frequently associated with some rodents in Europe, and *B. garinii* with some bird populations, whereas *B. burgdorferi* (*s.s.*) appear to be less specialized [40].

The identified *B. burgdorferi* (*s.l.*) genospecies are considered amongst the most important causative agents of LB in Europe and Asia [41–43]. These results are consistent with investigations reporting *I. ricinus*, the major vector of the causative agents of LB, as the main and most abundant tick species in the vegetation of the North of the Iberian Peninsula [19–21, 29, 44] since areas with abundant rainfall, large vegetation cover, abundant wildlife populations and grazing domestic animals provide suitable habitats for that tick species [45]. In contrast, *I. ricinus* is a tick of minor importance in drier areas of central and southern Spain where other species, such as *Hyalomma lusitanicum* and *Dermacentor marginatus,* are predominant [38, 44]; for this reason most of LB cases in Spain have been diagnosed in the northern regions [8, 46, 47].

*Borrelia miyamotoi*, related to the relapsing fever spirochete group, was only identified in four single ticks and in one *I. ricinus* male pool; this is the first report of this *Borrelia* species in free-living ticks from Spain. Despite the low percentages of *B. miyamotoi* detected in both the present study and in questing ticks from other European countries [48–51], it must be considered that this *Borrelia* species has been recently identified as a human pathogen, and it can be transmitted by all tick life stages [52]. Therefore, its presence should be taken into consideration in clinical patients.

Pooled testing is commonly used to estimate the presence of infection where the pathogen prevalence is likely low [53]. Our results also revealed that pooled testing is useful as a first approach when no epidemiological data on the pathogen is available in a particular area. In fact, higher numbers of *B. burgdorferi* (*s.l.*) genospecies were identified by means of pooled testing. Nevertheless, considering that pooled results underestimate the level of infection of a pathogen when its prevalence in a population is high, individual testing should be then performed in case of a significant number of positive pools.

## Conclusions

A significant prevalence of *B. burgdorferi* (*s.l.*) genospecies was detected in questing ticks from northwestern Spain. This percentage of infection is similar to those found in some central and northern European countries, although a lower presence of *B. afzelii* and *B. burgdorferi* (*s.s.*) was detected in the study area. These results, together with the high incidence of LB previously diagnosed in humans from the same area in the last decade [11] and the high seroprevalence of *B. burgdorferi* (*s.l.*) in roe deer [12] confirm that Galicia is one of the most at-risk regions for acquiring LB in Spain. In addition, the first identification of *B. miyamotoi* in free-living ticks from Spain should be taken into consideration in clinical patients. Further longitudinal epidemiological studies must be carried out in order to assess a possible relation between the presence of different *Borrelia* genospecies and the increase of the incidence of LB in this area. This information will be useful for achieving a more detailed location of risk areas for acquiring LB, leading to the optimization of the diagnosis and treatment of the disease.

## Additional file

Additional file 1: Table S1. Supplementary sequence information of all individual and pooled samples from *Ixodes ricinus* and identified as *Borrelia burgdorferi* (*s.l.*) or *Borrelia miyamotoi*. *Borrelia burgdorferi* (*s.l.*) isolates were characterized at both the flagellin (*fla*) gene and the *rrfA-rrlB* intergenic spacer region (IGS); *Borrelia miyamotoi* isolates were characterized at the flagellin (*fla*) and the glycerophosphodiester phosphodiesterase (*GlpQ*) genes. For each isolate and gene, the amplicon length and the closest matching sequences in GenBank are included. The GenBank number of unique partial sequences is also included. (DOCX 35 kb)
