# Supplementary material for: Molecular identification of Borrelia spirochetes in questing Ixodes ricinus from northwestern Spain
Source: Parasit Vectors. 2017 Dec 20;10:615. doi: 10.1186/s13071-017-2574-x (PMC5738910; doi:10.1186/s13071-017-2574-x)
Supplement: Additional file 1: Table S1. — Supplementary sequence information of all individual and pooled samples from Ixodes ricinus and identified as Borrelia burgdorferi (s.l.) or Borrelia miyamotoi. Borrelia burgdorferi (s.l.) isolates were characterized at both the flagellin (fla) gene and the rrfA-rrlB intergenic spacer region (IGS); Borrelia miyamotoi isolates were characterized at the flagellin (fla) and the glycerophosphodiester phosphodiesterase (GlpQ) genes. For each isolate and gene, the amplicon length and the closest matching sequences in GenBank are included. The GenBank number of unique partial sequences is also included. (DOCX 35 kb) [file 13071_2017_2574_MOESM1_ESM.docx]

**Additional file 1: Table S1. Supplementary sequence information of all individual and pooled samples from *Ixodes ricinus* and identified as *Borrelia burgdorferi (s.l.)* or *Borrelia miyamotoi*. *Borrelia burgdorferi* (*s.l.*) isolates were characterized at both the flagellin (*fla*) gene and the *rrfA-rrlB* intergenic spacer region (IGS); *Borrelia miyamotoi* isolates were characterized at the flagellin (*fla*) and the glycerophosphodiester phosphodiesterase (*GlpQ*) genes. For each isolate and gene, the amplicon length and the closest matching sequences in GenBank are included. The GenBank number of unique partial sequences is also included.**

|  |  |  | ***fla*** | | |  | **IGS** | | | | |
| --- | --- | --- | --- | --- | --- | --- | --- | --- | --- | --- | --- |
|  | **Sample** | **Sample type*** | **Closest GenBank acc. number** | **Percentage of identity** | **GenBank number** |  | **Closest GenBank acc. number** | | **Percentage of identity** | | **GenBank number** |
| ***Borrelia afzelii*** | | | | |  |  |  | | | | |
|  | Cav 14 | I/N | KU672564.1 | 328/328 (100%) |  |  | CP018262.1 | 179/179 (100%) | |  | |
|  | Coa 28 | I/N | KU672564.1 | 297/297 (100%) |  |  | CP018262.1 | 267/267 (100%) | |  | |
|  | Nav 26 | I/N | KU672564.1 | 329/329 (100%) |  |  | CP018262.1 | 271/271 (100%) | |  | |
|  | Rat 27 | I/N | KX646197.1 | 334/334 (100%) |  |  | CP018262.1 | 249/251 (99%) | | MG245787 | |
|  | Rtor 01 | I/F | KU672564.1 | 284/284 (100%) |  |  | CP018262.1 | 282/282 (100%) | |  | |
|  | Rtor 35 | I/M | KU672564.1 | 257/257 (100%) |  |  | CP018262.1 | 290/290 (100%) | |  | |
|  | Rtor 37 | I/N | KU672556.1 | 277/277 (100%) |  |  | CP018262.2 | 281/281 (100%) | |  | |
|  | COA2M | P/M | KU672564.1 | 355/355 (100%) |  |  | CP018262.1 | 281/281 (100%) | |  | |
|  | COA2N | P/N | KU672564.1 | 354/355 (99%) | MG245774 |  | CP018262.1 | 263/263 (100%) | |  | |
|  | PON3N | P/N | KU672564.1 | 335/335 (100%) |  |  | CP018262.1 | 285/285 (100%) | |  | |
|  |  |  |  |  |  |  |  |  | |  | |
| ***Borrelia burgdorferi* s.s.** | | | | |  |  |  | | | | |
|  | NAV2N | P/N | KX646200.1 | 291/291 (100%) |  |  | CP019767.1 | 251/251 (100%) | |  | |
|  |  |  |  |  |  |  |  |  | |  | |
| ***Borrelia garinii*** | | | | |  |  |  | | | | |
|  | Beg 01 | I/F | KU672556.1 | 320/321 (99%) |  |  | CP018744.1 | 278/281 (99%) | | MG356949 | |
|  | Beg 10 | I/M | KU672556.1 | 328/328 (100%) |  |  | CP018744.1 | 290/290 (100%) | |  | |
|  | Beg 15 | I/N | KX646202.1 | 314/314 (100%) |  |  | CP018744.1 | 278/281 (99%) | |  | |
|  | Beg 19 | I/F | KF422825.1 | 322/322 (100%) |  |  | CP018744.1 | 289/292 (99%) | |  | |
|  | Cav 03 | I/F | AB091807.1 | 328/328 (100%) |  |  | CP018744.1 | 259/259 (100%) | |  | |
|  | Cav 12 | I/N | KX646202.1 | 306/306 (100%) |  |  | CP018744.1 | 280/284 (99%) | |  | |
|  | Cav 21 | I/N | KX646202.1 | 306/306 (100%) |  |  | CP018744.1 | 286/288 (99%) | | MG356955 | |
|  | Cav 23 | I/N | AB091807.1 | 305/305 (100%) |  |  | CP018744.1 | 286/289 (99%) | |  | |
|  | Co 02 | I/F | AB091807.1 | 306/306 (100%) |  |  | CP018744.1 | 289/289 (99%) | |  | |
|  | Co 03 | I/F | KJ577820.1 | 299/299 (100%) |  |  | CP018744.1 | 290/293 (99%) | | MG356956 | |
|  | Co 06 | I/M | AB091807.1 | 322/322 (100%) |  |  | CP018744.1 | 285/288 (99%) | |  | |
|  | Co 12 | I/M | KJ577820.1 | 306/306 (100%) |  |  | CP018744.1 | 285/288 (99%) | |  | |
|  | Co 15 | I/M | KJ577820.1 | 263/263 (100%) |  |  | CP018744.1 | 290/290 (100%) | |  | |
|  | Nav 03 | I/F | KX646202.1 | 277/277 (100%) |  |  | CP018744.1 | 286/288 (99%) | |  | |
|  | Nav 04 | I/F | KU672556.1 | 324/324 (100%) |  |  | CP018744.1 | 288/288 (100%) | |  | |
|  | Pont 04 | I/F | KR782233.1 | 327/327 (100%) |  |  | CP018744.1 | 289/289 (100%) | |  | |
|  | Pont 05 | I/F | KU672556.1 | 319/320 (99%) |  |  | CP018744.1 | 290/290 (100%) | |  | |
|  | Rat 13 | I/N | KU672556.1 | 338/339 (99%) |  |  | CP018744.1 | 277/284 (98%) | | MG356951 | |
|  | Rat 20 | I/N | KF422825.1 | 321/321 (100%) |  |  | CP018744.1 | 281/281 (100%) | |  | |
|  | Rat 21 | I/F | KX646202.1 | 325/325 (100%) |  |  | CP018744.1 | 290/293 (99%) | | MG356954 | |
|  | Rat 22 | I/M | KX646202.1 | 299/299 (100%) |  |  | CP018744.1 | 280/284 (99%) | | MG356950 | |
|  | Rat 36 | I/M | KU672556.1 | 325/326 (99%) |  |  | CP018744.1 | 283/285 (99%) | |  | |
|  | Rtor 03 | I/F | KJ577820.1 | 304/304 (100%) |  |  | CP018744.1 | 284/284 (100%) | |  | |
|  | Rtor 04 | I/F | KJ577820.1 | 284/284 (100%) |  |  | CP018744.1 | 289/292 (99%) | |  | |
|  | Rtor 05 | I/F | KX646202.1 | 305/305 (100%) |  |  | CP018744.1 | 285/288 (99%) | |  | |
|  | Rtor 18 | I/N | AB091807.1 | 305/305 (100%) |  |  | CP018744.1 | 290/290 (100%) | |  | |
|  | Rtor 31 | I/N | KU672556.1 | 159/160 (99%) |  |  | KX906940.1 | 231/233 (99%) | | MG356953 | |
|  | Rtor 37 | I/N | KU672556.1 | 277/277 (100%) |  |  | CP018744.1 | 276/281 (98%) | | MG356952 | |
|  | Vb32 | I/F | KX646202.1 | 323/323 (100%) |  |  | CP018744.1 | 281/281 (100%) | |  | |
|  | Vil 02 | I/F | KU672556.1 | 306/306 (100%) |  |  | CP018744.1 | 290/290 (100%) | |  | |
|  | BEG1H | P/F | KX646202.1 | 335/335 (100%) |  |  | CP018744.1 | 276/278 (99%) | |  | |
|  | BEG1M | P/M | KX646202.1 | 342/342 (100%) |  |  | CP018744.1 | 285/288 (99%) | |  | |
|  | BEG2H | P/F | KF422825.1 | 337/337 (100%) |  |  | CP018744.1 | 290/293 (99%) | |  | |
|  | BEG2N | P/N | KX646202.1 | 342/342 (100%) |  |  | CP018744.1 | 286/289 (99%) | |  | |
|  | BEG3H | P/F | KU672556.1 | 341/342 (99%) |  |  | CP018744.1 | 262/264 (99%) | |  | |
|  | BEG3N | P/N | KX646202.1 | 354/355 (99%) |  |  | CP018744.1 | 279/281 (99%) | |  | |
|  | BEG4H | P/F | KX646202.1 | 342/342 (100%) |  |  | CP018744.1 | 290/293 (99%) | |  | |
|  | BEG4M | P/M | KU672556.1 | 342/342 (100%) |  |  | CP018744.1 | 286/286 (100%) | |  | |
|  | BEG5M | P/M | KF422825.1 | 355/356 (99%) | MG245780 |  | CP018744.1 | 283/286 (99%) | |  | |
|  | CAS1N1 | P/N | KX646202.1 | 324/324 (100%) |  |  | CP018744.1 | 285/288 (99%) | |  | |
|  | CAS1N5 | P/N | KX646202.1 | 327/327 (100%) |  |  | CP018744.1 | 278/281 (99%) | |  | |
|  | CER1M | P/M | KU672556.1 | 354/356 (99%) |  |  | CP018744.1 | 279/279 (100%) | |  | |
|  | CER1N2 | P/N | KU672556.1 | 354/355 (99%) |  |  | CP018744.1 | 279/279 (100%) | |  | |
|  | CER1N3 | P/N | KR782233.1 | 354/355 (99%) |  |  | CP018744.1 | 278/281 (99%) | |  | |
|  | COAN3 | P/N | KU672556.1 | 346/347 (99%) |  |  | CP018744.1 | 290/293 (99%) | |  | |
|  | COAN4 | P/N | KF422825.1 | 266/267 (99%) |  |  | CP018744.1 | 286/289 (99%) | |  | |
|  | COS1M | P/M | KF894061.1 | 353/355 (99%) | MG245784 |  | CP018744.1 | 286/289 (99%) | |  | |
|  | COS1N | P/N | KF990322.1 | 342/342 (100%) |  |  | CP018744.1 | 287/287 (100%) | |  | |
|  | COS2M | P/M | KJ577820.1 | 342/342 (100%) |  |  | CP018744.1 | 287/287 (100%) | |  | |
|  | COS4N | P/N | KF422825.1 | 353/354 (99%) |  |  | CP018744.1 | 285/287 (99%) | |  | |
|  | FON5H | P/F | KX646202.1 | 330/330 (100%) |  |  | CP018744.1 | 245/247 (99%) | |  | |
|  | FON8 | P/N | KX646202.1 | 334/334 (100%) |  |  | CP018744.1 | 278/281 (99%) | |  | |
|  | LUG1H | P/F | KX646202.1 | 337/337 (100%) |  |  | CP018744.1 | 281/281 (100%) | |  | |
|  | LUG1N | P/N | KU672556.1 | 345/346 (99%) |  |  | CP018744.1 | 278/281 (99%) | |  | |
|  | LUG2N | P/N | KU672556.1 | 354/355 (99%) | MG245786 |  | CP018744.1 | 278/278 (100%) | |  | |
|  | LUG4N | P/N | KU672556.1 | 354/356 (99%) | MG245785 |  | CP018744.1 | 286/289 (99%) | |  | |
|  | LUG6N | P/N | KF422825.1 | 334/335 (99%) |  |  | CP018744.1 | 245/247 (99%) | |  | |
|  | MEI1N | P/N | KX646202.1 | 330/330 (100%) |  |  | CP018744.1 | 289/292 (99%) | |  | |
|  | MEI2N | P/N | KX646202.1 | 354/355 (99%) | MG245779 |  | CP018744.1 | 286/286 (100%) | |  | |
|  | MEI3N | P/N | KJ577820.1 | 346/347 (99%) |  |  | CP018744.1 | 278/281 (99%) | |  | |
|  | NAV1N | P/N | KX646202.1 | 335/335 (100%) |  |  | CP018744.1 | 289/292 (99%) | |  | |
|  | NAV2H | P/F | KU672556.1 | 330/330 (100%) |  |  | CP018744.1 | 253/256 (99%) | |  | |
|  | NAV3H | P/F | KX646202.1 | 341/341 (100%) |  |  | CP018744.1 | 282/285 (99%) | |  | |
|  | NAV3M | P/M | KX646198.1 | 335/336 (99%) | MG245782 |  | CP018744.1 | 278/281 (99%) | |  | |
|  | NAV3N | P/N | KU672556.1 | 350/352 (99%) |  |  | CP018744.1 | 285/288 (99%) | |  | |
|  | NAV4N | P/N | KJ577820.1 | 346/347 (99%) | MG245783 |  | CP018744.1 | 286/289 (99%) | |  | |
|  | PON7H | P/F | KU672556.1 | 340/342 (99%) |  |  | CP018744.1 | 273/273 (100%) | |  | |
|  | RIO1N | P/N | KF990320.1 | 330/330 (100%) |  |  | CP018744.1 | 289/289 (100%) | |  | |
|  | RIO2H | P/F | KX646202.1 | 337/338 (99%) |  |  | CP018744.1 | 290/290 (100%) | |  | |
|  | RIO2M | P/M | KR782233.1 | 332/333 (99%) | MG245781 |  | CP018744.1 | 285/285 (100%) | |  | |
|  | RIO2N | P/N | KF422825.1 | 342/343 (99%) |  |  | CP018744.1 | 284/284 (100%) | |  | |
|  | RIO3H | P/F | KU672556.1 | 331/331 (100%) |  |  | CP018744.1 | 281/281 (100%) | |  | |
|  | RIO3N | P/N | KF990320.1 | 343/343 (100%) |  |  | CP018744.1 | 290/293 (99%) | |  | |
|  | RIO5N | P/N | KF422825.1 | 342/343 (99%) |  |  | CP018744.1 | 286/289 (99%) | |  | |
|  | VIL2H | P/F | KU672556.1 | 330/330 (100%) |  |  | CP018744.1 | 290/293 (99%) | |  | |
|  | VLL2H | P/F | KX646202.1 | 342/342 (100%) |  |  | CP018744.1 | 251/253 (99%) | |  | |
|  |  |  |  |  |  |  |  |  | |  | |
| ***Borrelia lusitaniae*** | | | | |  |  |  | | | | |
|  | Fons 03 | I/F | KR782243.1 | 268/268 (100%) |  |  | EU078961.1 | 251/251 (100%) | |  | |
|  | Fons 10 | I/M | KR782238.1 | 276/276 (100%) |  |  | AY209179.2 | 232/232 (100%) | |  | |
|  | Pont 11 | I/F | KR782238.1 | 306/306 (100%) |  |  | AY209179.2 | 186/186 (100%) | |  | |
|  | Pont 12 | I/F | KR782238.1 | 264/264 (100%) |  |  | EU078961.1 | 263/264 (99%) | | MG245789 | |
|  | Fons 15 | I/N | KX646194.1 | 326/326 (100%) |  |  | AY209179.2 | 235/238 (99%) | | MG245788 | |
|  | Fons 28 | I/F | KX646194.1 | 304/304 (100%) |  |  | AY209179.2 | 264/264 (100%) | |  | |
|  | Rat 06 | I/M | KX646194.1 | 326/326 (100%) |  |  | AY209179.2 | 239/239 (100%) | |  | |
|  | Vil 05 | I/M | KX646194.1 | 316/316 (100%) |  |  | AY209179.2 | 251/251 (100%) | |  | |
|  | PON1H | P/F | KX646194.1 | 328/328 (100%) |  |  | AY209179.2 | 271/271 (100%) | |  | |
|  | PON2H | P/F | KR782243.1 | 353/355 (99%) | MG245773 |  | EU078961.1 | 246/246 (100%) | |  | |
|  | PON3H | P/F | KX646194.1 | 343/344 (99%) |  |  | AY209179.2 | 253/253 (100%) | |  | |
|  | PON5H | P/F | KR782238.1 | 327/327 (100%) |  |  | EU078961.1 | 244/245 (99%) | |  | |
|  | PON6H | P/F | KR782238.1 | 332/332 (100%) |  |  | EU078961.1 | 259/259 (100%) | |  | |
|  | PON2N | P/N | KX646194.1 | 343/344 (99%) |  |  | AY209179.2 | 264/264 (100%) | |  | |
|  | PON4N | P/N | KX646194.1 | 344/345 (99%) | MG245772 |  | AY209179.2 | 263/263 (100%) | |  | |
|  |  |  |  |  |  |  |  |  | |  | |
| ***Borrelia valaisiana*** | | | | |  |  |  | | | | |
|  | Beg 13 | I/N | KX646197.1 | 326/326 (100%) |  |  | CP009117.1 | 278/279 (99%) | |  | |
|  | Cav 06 | I/F | KX646197.1 | 323/323 (100%) |  |  | CP009117.1 | 289/289 (100%) | |  | |
|  | Cav 07 | I/F | KX646197.1 | 325/325 (100%) |  |  | CP009117.1 | 290/291 (99%) | |  | |
|  | Cav 25 | I/N | KX646197.1 | 333/333 (100%) |  |  | CP009117.1 | 279/279 (100%) | |  | |
|  | Co 05 | I/F | KX646197.1 | 241/241 (100%) |  |  | CP009117.1 | 289/289 (100%) | |  | |
|  | Co 07 | I/M | KX646197.1 | 302/302 (100%) |  |  | CP009117.1 | 289/289 (100%) | |  | |
|  | Co 13 | I/F | KX646197.1 | 321/321 (100%) |  |  | CP009117.1 | 251/252 (99%) | |  | |
|  | Nav 05 | I/F | HM345912.1 | 321/322 (99%) |  |  | CP009117.1 | 291/291 (100%) | |  | |
|  | Nav 17 | I/N | KX646197.1 | 332/332 (100%) |  |  | CP009117.1 | 283/283 (100%) | |  | |
|  | Rat 02 | I/F | KX646197.1 | 302/302 (100%) |  |  | CP009117.1 | 275/275 (100%) | |  | |
|  | Rat 07 | I/M | KX646197.1 | 267/267 (100%) |  |  | CP009117.1 | 290/290 (100%) | |  | |
|  | Rtor 28 | I/N | KX646197.1 | 328/328 (100%) |  |  | CP009117.1 | 284/284 (100%) | |  | |
|  | Vb33 | I/F | HM345912.1 | 327/328 (99%) |  |  | CP009117.1 | 264/264 (100%) | |  | |
|  | Vb36 | I/M | KF990324.1 | 328/328 (100%) |  |  | CP009117.1 | 283/283 (100%) | |  | |
|  | Vb39 | I/N | KX646197.1 | 314/314 (100%) |  |  | CP009117.1 | 287/287 (100%) | |  | |
|  | Vil 07 | I/N | HM345912.1 | 342/343 (100%) |  |  | CP009117.1 | 286/286 (100%) | |  | |
|  | Vil 08 | I/N | HM345912.1 | 239/240 (99%) |  |  | CP009117.1 | 291/292 (99%) | | MG245790 | |
|  | BEG1N | P/N | KF990324.1 | 354/355 (99%) | MG245775 |  | CP009117.1 | 286/286 (100%) | |  | |
|  | BEG5N | P/N | KF990324.1 | 343/344 (99%) |  |  | CP009117.1 | 287/288 (99%) | |  | |
|  | CAS1H2 | P/F | KF990324.1 | 354/355 (99%) |  |  | CP009117.1 | 285/285 (100%) | |  | |
|  | CAS1N3 | P/N | KF990324.1 | 351/352 (99%) |  |  | CP009117.1 | 252/252 (100%) | |  | |
|  | CAS1N4 | P/N | KF990324.1 | 341/342 (99%) |  |  | CP009117.1 | 291/291 (100%) | |  | |
|  | CER1N5 | P/N | HM345912.1 | 341/342 (99%) | MG245777 |  | CP009117.1 | 283/283 (100%) | |  | |
|  | COAH1 | P/F | KX646197.1 | 330/330 (100%) |  |  | CP009117.1 | 291/291 (100%) | |  | |
|  | COAH2 | P/F | KF990324.1 | 345/346 (99%) |  |  | CP009117.1 | 284/284 (100%) | |  | |
|  | COS1H | P/F | KF990324.1 | 348/349 (99%) |  |  | CP009117.1 | 289/289 (100%) | |  | |
|  | COS2H | P/F | KF990324.1 | 330/330 (100%) |  |  | CP009117.1 | 286/287 (99%) | |  | |
|  | LUG1M | P/M | KX646197.1 | 333/333 (100%) |  |  | CP009117.1 | 268/268 (100%) | |  | |
|  | LUG2H | P/F | KF990324.1 | 354/355 (99%) |  |  | CP009117.1 | 286/286 (100%) | |  | |
|  | LUG3H | P/F | KF990324.1 | 345/346 (99%) |  |  | CP009117.1 | 290/290 (100%) | |  | |
|  | LUG3N | P/N | KX646197.1 | 346/348 (99%) |  |  | CP009117.1 | 287/287 (100%) | |  | |
|  | LUG5N | P/N | HM345912.1 | 340/342 (99%) | MG245778 |  | CP009117.1 | 289/289 (100%) | |  | |
|  | NAV1M | P/M | KX646197.1 | 340/342 (99%) | MG245776 |  | CP009117.1 | 262/262 (100%) | |  | |
|  | PON1N | P/N | KF990324.1 | 328/328 (100%) |  |  | CP009117.1 | 266/266 (100%) | |  | |
|  | PON4H | P/F | KF990324.1 | 354/355 (99%) |  |  | CP009117.1 | 289/289 (100%) | |  | |
|  | RIO1H | P/F | KF990324.1 | 354/355 (99%) |  |  | CP009117.1 | 283/284 (99%) | |  | |
|  | RIO4N | P/N | KF990324.1 | 343/343 (100%) |  |  | CP009117.1 | 283/283 (100%) | |  | |
|  | VIL3 | P/N | KF990324.1 | 353/354 (99%) |  |  | CP009117.1 | 285/285 (100%) | |  | |
|  | VIL4 | P/N | KX646197.1 | 353/355 (99%) |  |  | CP009117.1 | 289/289 (100%) | |  | |
|  | VIL5 | P/N | KF990324.1 | 354/355 (99%) |  |  | CP009117.1 | 285/285 (100%) | |  | |
|  | VLL1H | P/F | HM345912.1 | 340/342 (99%) |  |  | CP009117.1 | 266/266 (100%) | |  | |
|  | VLL1N | P/N | KF990324.1 | 341/343 (99%) |  |  | CP009117.1 | 278/278 (100%) | |  | |
|  | VLL2M | P/M | KF990324.1 | 354/355 (99%) |  |  | CP009117.1 | 276/276 (100%) | |  | |
|  | VLL4N | P/N | KF990324.1 | 354/355 (99%) |  |  | CP009117.1 | 256/256 (100%) | |  | |
|  | VLL5N | P/N | KF990324.1 | 348/349 (99%) |  |  | CP009117.1 | 284/284 (100%) | |  | |
|  |  |  |  |  |  |  |  |  | |  | |
|  |  |  |  |  |  |  |  |  | |  | |
|  |  |  | ***fla*** | |  |  | **GlpQ** | | | | |
| ***Borrelia miyamotoi*** | | | | |  |  |  | | | | |
|  | Fons 05 | I/F | KT932823.1 | 243/243 (100%) |  |  | AB824855.1 | 846/846 (100%) | |  | |
|  | Nav 09 | I/M | KT932823.1 | 321/321 (100%) |  |  | AB824855.1 | 828/828 (100%) | |  | |
|  | Rtor 14 | I/N | KT932823.1 | 253/253 (100%) |  |  | AB824855.1 | 845/845 (100%) | |  | |
|  | Vb 11 | I/N | KT932823.1 | 321/321 (100%) |  |  | AB824855.1 | 843/843 (100%) | |  | |
|  | NAV2M | P/M | KT932823.1 | 325/325 (100%) |  |  | AB824855.1 | 836/836 (100%) | |  | |

*P: pooled sample; I: individual sample; N: nymph; M: adult male; F: adult female
